# Chronic High Fat Diet Intake Impairs Hepatic Metabolic Parameters in Ovariectomized Sirt3 KO Mice

**DOI:** 10.3390/ijms22084277

**Published:** 2021-04-20

**Authors:** Marija Pinterić, Iva I. Podgorski, Marijana Popović Hadžija, Ivana Tartaro Bujak, Ana Tadijan, Tihomir Balog, Sandra Sobočanec

**Affiliations:** 1 Division of Molecular Medicine, Ruđer Bošković Institute, 10000 Zagreb, Croatia; mpinter@irb.hr (M.P.); iskrinj@irb.hr (I.I.P.); mhadzija@irb.hr (M.P.H.); Ana.Tadijan@irb.hr (A.T.); balog@irb.hr (T.B.); 2 Division of Materials Chemistry, Ruđer Bošković Institute, 10000 Zagreb, Croatia; itartaro@irb.hr

**Keywords:** sirtuin 3, ovariectomy, high fat diet, fatty liver

## Abstract

High fat diet (HFD) is an important factor in the development of metabolic diseases, with liver as metabolic center being highly exposed to its influence. However, the effect of HFD-induced metabolic stress with respect to ovary hormone depletion and sirtuin 3 (Sirt3) is not clear. Here we investigated the effect of Sirt3 in liver of ovariectomized and sham female mice upon 10 weeks of feeding with standard-fat diet (SFD) or HFD. Liver was examined by Folch, gas chromatography and lipid hydroperoxide analysis, histology and oil red staining, RT-PCR, Western blot, antioxidative enzyme and oxygen consumption analyses. In SFD-fed WT mice, ovariectomy increased Sirt3 and fatty acids synthesis, maintained mitochondrial function, and decreased levels of lipid hydroperoxides. Combination of ovariectomy and Sirt3 depletion reduced *pparα*, Scd-1 ratio, MUFA proportions, CII-driven respiration, and increased lipid damage. HFD compromised CII-driven respiration and activated peroxisomal ROS scavenging enzyme catalase in sham mice, whereas in combination with ovariectomy and Sirt3 depletion, increased body weight gain, expression of NAFLD- and oxidative stress-inducing genes, and impaired response of antioxidative system. Overall, this study provides evidence that protection against harmful effects of HFD in female mice is attributed to the combined effect of female sex hormones and Sirt3, thus contributing to preclinical research on possible sex-related therapeutic agents for metabolic syndrome and associated diseases.

## 1. Introduction

The metabolic syndrome is a cluster of risk factors responsible for the development of cardiovascular diseases and many other health problems, and as such is one of the leading risks for global deaths representing a serious threat to public health [1]. A high fat diet (HFD) is an important factor in the development of many metabolic diseases, with liver as a metabolic center being highly exposed to its influence [2]. Metabolic syndrome can be effectively mimicked and studied in rodent models using various dietary interventions, including HFD [3], which then lead to mitochondrial dysfunction and other metabolic changes induced by oxidative stress (reviewed in [1]). Feeding mice with HFD results in one of the diet-induced models of non-alcoholic fatty liver disease (NAFLD), which is accompanied by liver inflammation and steatosis [4]. Indeed, hepatic steatosis occurs when high concentrations of circulatory fatty acids (FAs) reaching the liver and de novo lipogenesis are not counterbalanced by FA oxidation or lipid export as lipoproteins [5].

In most mammals, including humans, life expectancy is female-biased [6,7]. Females show lower incidence of some age-related pathologies linked with oxidative stress and this sex-difference disappears after menopause, which leads to the conclusion that this protection is attributed to sex hormones (reviewed in [8]). Thus, one approach to study age-linked pathologies is to investigate hormone-depleted or -augmented animals and their defense from metabolic stressors. Estradiol (E2) is an important regulator of energy homeostasis, thus making it a potential target for preventing or treating metabolic disorders. Previous studies established the association of metabolic syndrome and E2 loss during menopause in women (reviewed in [9]) and reported that E2 can alter hepatic proteins involved in de novo lipid synthesis [10]. However, the mechanism behind those observations, especially how the fat-lowering action of E2 is modulated in the liver, remains elusive, especially in a sex-related manner.

Sirtuin 3 (Sirt3) is a mitochondrial protein that integrates cellular energy metabolism and plays an important role in preventing metabolic syndrome [4]. Although Sirt4 and Sirt5 are also present in mitochondria, Sirt3 is the main mitochondrial deacetylase because only Sirt3-knockout mice show hyperacetylation of mitochondrial proteins and less effective mitochondria. In addition, Sirt3 is involved in the regulation of all mitochondrial functions, including the tricarboxylic acid (TCA), the urea cycle, amino acid metabolism, fatty acid oxidation, oxidative phosphorylation (OXPHOS), ROS detoxification, mitochondrial dynamics, and the mitochondrial unfolded protein response (UPR) [11,12]. It promotes mitochondrial oxidative metabolism via deacetylation of numerous metabolic enzymes, including those involved in FA catabolism, as demonstrated earlier by an abnormal accumulation of FA oxidation intermediates in Sirt3 KO mice [4]. It was also shown that Sirt3 expression is reduced during chronic HFD in male mice [4,13]. Although E2-dependent protection includes improvement of mitochondrial function [14], it is not clear whether Sirt3, as a pivotal factor regulating mitochondrial biogenesis and reactive oxygen species (ROS) management, participates in these events.

In our recent study, we found significant sex differences in mice at the level of metabolic, oxidant, antioxidant, and mitochondrial parameters upon HFD. Also, we pointed towards a different role of Sirt3 in males and females under the conditions of nutritive stress, with higher reliance of males than females to the effect of Sirt3 against HFD-induced metabolic dysregulation [13]. These observations led us to the hypothesis that females’ protection from HFD-induced metabolic dysregulation in vivo could be attributed to the complementary beneficial effect of Sirt3 and ovary hormones. However, the mechanism by which this combination operates still needs to be elucidated. Therefore, this study explored the metabolic, mitochondrial, oxidative, and antioxidative parameters following HFD and ovarian hormone deprivation in young adult Sirt3 WT and Sirt3 KO female mice.

## 2. Results

### 2.1. Ovariectomy Increases Sirt3 and pgc1-α Expression in the Liver of Female Mice

**Figure 1:**
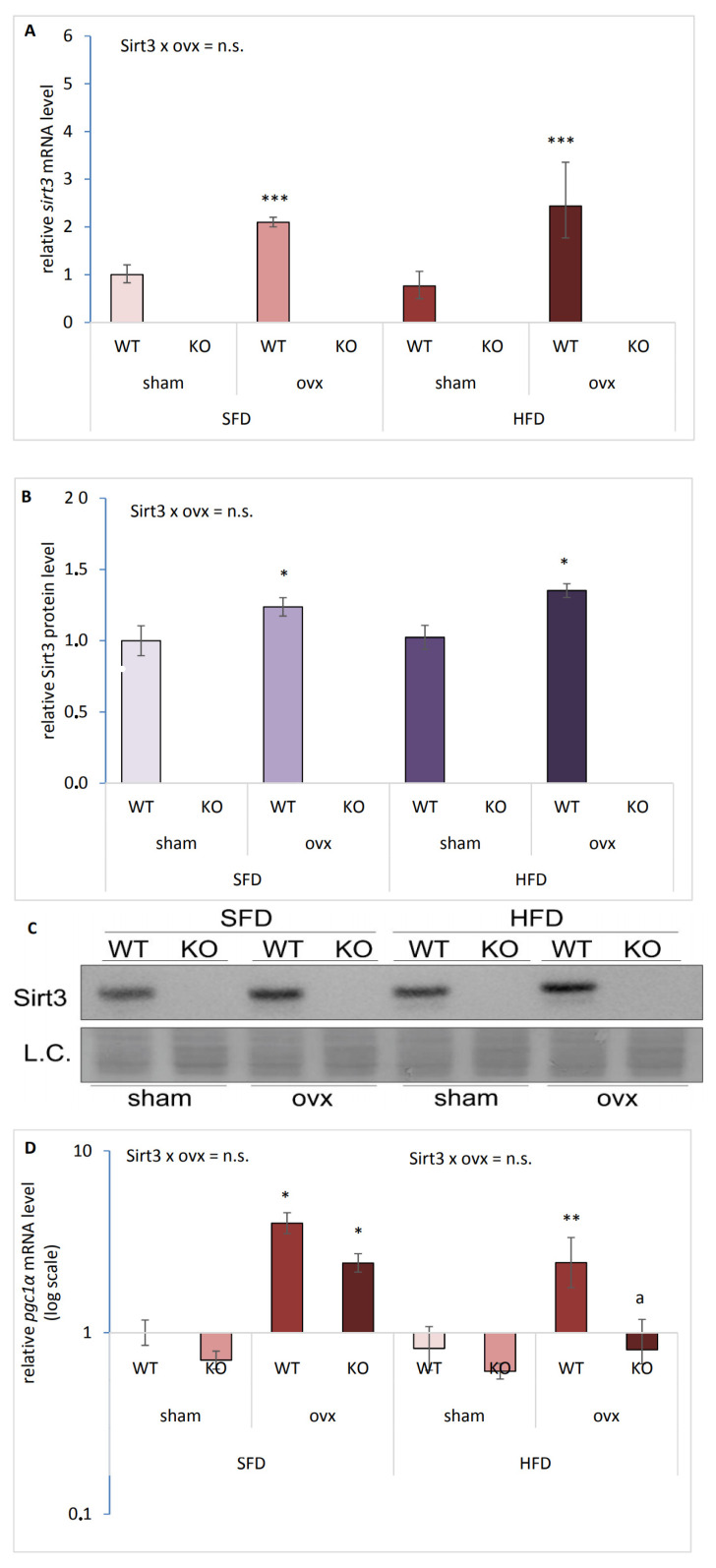
Ovariectomy (ovx) increases Sirt3 and *pgc1-α* in liver of female mice. (**A**) *sirt3* gene expression in sham and ovx Sirt3 WT and KO mice. SFD: WT sham vs. ovx (*** *p* < 0.001). HFD: WT sham vs. ovx (*** *p* < 0.001). SFD vs. HFD: no changes. (**B**) Graphical display of averaged densitometry values for Sirt3 protein expression in sham and ovx Sirt3 WT and KO mice. SFD: WT sham vs. ovx (* *p* < 0.05). HFD: WT sham vs. ovx (* *p* < 0.05). SFD vs. HFD: no changes. (**C**) Immunoblot of Sirt3 protein expression. Amidoblack was used as a loading control (L.C.). (**D**) *pgc1-α* gene expression in sham and ovx Sirt3 WT and KO mice. SFD: sham vs. ovx (* *p* <0.05). HFD: WT sham vs. ovx (** *p* < 0.01); ovx WT vs. KO (^a^ *p* < 0.01). SFD vs. HFD: no changes. Data are shown as mean ± SD. *n* = 3 mice per group. The representative image is displayed.

Since ovaries are the main source of female sex hormones’ production in the body [15], we performed ovariectomy (ovx) to assess the effect of ovary hormones in our experiments. Loss of ovarian hormones was confirmed by reduced uterus size and by cytological examination of vaginal smears, showing estrous phase in control (sham) and anestrous phase in ovx mice (Supplemental Figure S1A–C). To assess the functional role of hepatic Sirt3 with respect to ovx and HFD, we examined gene and protein expression in female sham and ovx Sirt3 WT and KO mice after 10 weeks of feeding with SFD or HFD. Ovx increased *sirt3* gene (*** *p* < 0.001) and protein (* *p* < 0.05) expression in both SFD and HFD conditions (Figure 1A–C). Thus, ovx upregulated Sirt3 irrespective of type of diet in WT female mice. A similar pattern was observed with peroxisome proliferator-activated receptor-gamma coactivator-1 alpha (*pgc1-α*), a master regulator of mitochondrial function [16]. Ovx upregulated *pgc1-α* gene expression regardless of Sirt3 in SFD conditions (* *p* < 0.05) (Figure 1D). Following HFD, KO normalized ovx-induced (** *p* < 0.01) *pgc1-α* gene expression (^a^ *p* < 0.01). These data indicate that ovx induces both *pgc1-α* and Sirt3 in WT mice irrespective of diet and that expression of the *pgc1-α* in ovx KO mice depends on the type of diet.

### 2.2. The Effect of Sirt3 and Ovx on Body Weight Gain Depends on the Type of Diet

**Figure 2:**
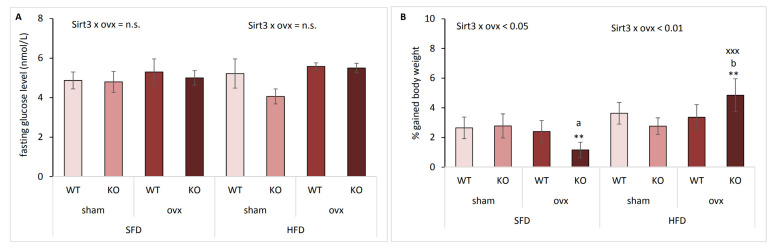
The effect of Sirt3 and ovariectomy (ovx) on body weight gain is diet dependent. (**A**) Fasting blood glucose in sham and ovx Sirt3 WT and KO mice. SFD: no changes. HFD: no changes. SFD vs. HFD: no changes. (**B**) Gained body weight (%) in sham and ovx Sirt3 WT and KO mice. SFD: ovx WT vs. KO (^a^ *p* < 0.05); KO sham vs. ovx (** *p* <0.01). HFD: ovx WT vs. KO (^b^ *p* < 0.01); KO sham vs. ovx (** *p* < 0.01). SFD vs. HFD: KO ovx (^xxx^ *p* < 0.001). Data are shown as mean ± SD. *n* = 6 mice per group.

Fasting glucose level remained unchanged by either ovx or Sirt3 depletion in both SFD and HFD-fed mice (Figure 2A). In agreement with our previous observations [13], we found no change in body weight in SFD- and HFD-fed sham mice (data not shown). However, body weight gain was affected differently in ovx KO mice, depending on type of diet: SFD-fed KO ovx mice gained less weight than WT ovx (^a^ *p* < 0.05) or sham KO mice (** *p* < 0.01) (Figure 2B). Contrary to SFD conditions, upon HFD, KO ovx mice gained more weight than WT ovx (^b^ *p* < 0.01) or sham KO mice (** *p* < 0.01). Also, HFD-fed ovx KO mice were the only group that had increased body weight gain compared to their SFD littermates (^xxx^ *p* < 0.001). These data indicate that ovary hormone deficiency in the absence of Sirt3 makes these mice most resistant towards gaining weight on SFD but also most sensitive towards gaining weight upon HFD.

### 2.3. Sirt3 and Ovx have Combined Effect on the Expression of Genes Responsible for Lipid Metabolism and Oxidative Stress

**Figure 3:**
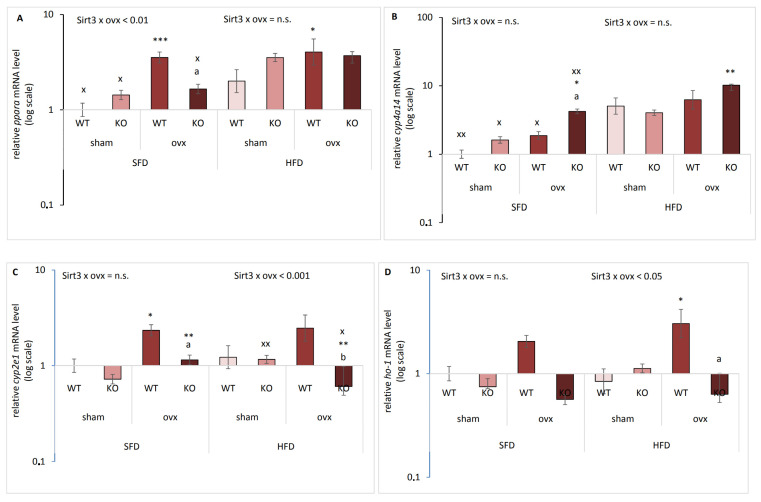
Sirt3 and ovx have a combined effect on the expression of genes involved in lipid metabolism and oxidative stress. Graphical displays of gene expression levels in sham and ovx Sirt3 WT and KO mice after 10 weeks of feeding with SFD or HFD. (**A**) *pparα*. SFD: ovx WT vs. KO (^a^ *p* < 0.01); WT sham vs. ovx (*** *p* < 0.001). HFD: WT sham vs. ovx (* *p* < 0.05). SFD vs. HFD: sham (^x^ *p* < 0.05), ovx KO (^x^ *p* < 0.05). (**B**) *cyp4a14*. SFD: ovx WT vs. KO (^a^ *p* < 0.05). HFD: KO sham vs. ovx (** *p* < 0.01). SFD vs. HFD: sham WT (^xx^ *p* < 0.01); sham KO, ovx WT (^x^ *p* < 0.05); ovx KO (^xx^ *p* < 0.01). (**C**) *cyp2e1*. SFD: ovx WT vs. KO (^a^ *p* < 0.01); WT sham vs. ovx (* *p* < 0.05); KO sham vs. ovx (** *p* < 0.01). HFD: ovx WT vs. KO (^b^ *p* < 0.01); KO sham vs. ovx (** *p* < 0.01). SFD vs. HFD: sham KO (^xx^ *p* < 0.01); ovx KO (^x^ *p* < 0.05). (**D**) *ho-1*. SFD: no changes. HFD: WT sham vs. ovx (* *p* < 0.05); ovx WT vs. KO (^a^ *p* < 0.05). SFD vs. HFD: no changes. β-actin was used for normalization. Data are shown as mean ± SD. *n* = 3 mice per group in technical triplicates.

Due to significant differences in body weight gain with respect to Sirt3 and ovx in SFD and HFD, we investigated whether combination of Sirt3 depletion and ovx affected genes involved in the lipid metabolism and oxidative stress, i.e., peroxisome proliferator-activated receptor alpha (*pparα*) [17], cytochrome P450 4a14 (*cyp4a14*)*, cyp2e1* [18], and heme oxygenase-1 (*ho-1*) [19]. In SFD-fed mice, ovx affected *pparα* expression differently depending on Sirt3: ovx increased *pparα* only in WT mice (*** *p* < 0.001), without affecting KO mice (^a^ *p* < 0.01) (Figure 3A). In HFD-fed mice, *pparα* was also increased only in ovx WT compared to sham WT mice (* *p* < 0.05). HFD generally increased levels of *pparα* in all groups (^x^ *p* < 0.05) except in ovx WT. In SFD conditions, *cyp4a14* expression was increased in ovx KO mice compared to both ovx WT (^a^ *p* < 0.01) and sham KO mice (* *p* < 0.05) (Figure 3B). In HFD-fed conditions, ovx increased *cyp4a14* expression only in KO mice (^**^ *p* < 0.001). Also, *cyp4a14* expression was higher in all HFD-fed mice compared to the respective SFD-fed groups (^x^ *p* < 0.05, ^xx^ *p* < 0.01). In SFD-fed mice, ovx increased *cyp2e1* expression in both WT (* *p* < 0.05) and KO mice (** *p* < 0.01) (Figure 3C). Sirt3 depletion reverted upregulated *cyp2e1* expression in ovx mice (^a^ *p* < 0.05). Changes in *cyp2e1* expression between SFD and HFD-fed mice were observed in sham KO (^xx^ *p* < 0.01) and ovx KO group (^x^ *p* < 0.05), whereas HFD increased or decreased *cyp2e1* in these groups, respectively. *Ho-1* gene expression level remained unchanged in SFD conditions (Figure 3D). Following HFD, in WT mice ovx increased *ho-1* gene expression level compared to WT sham mice (* *p* < 0.05), but Sirt3 depletion reverted it (^a^ *p* < 0.05). These data indicate that ovx induces *pparα*, *cyp2e1*, and *ho-1* genes in WT mice, and Sirt3 depletion mostly reverses this effect. On the other hand, *cyp4a14* is induced by HFD, and additionally by the combination of ovx and Sirt3 depletion.

### 2.4. Sirt3 KO Ovx Mice Have Reduced Lipid Accumulation in SFD Conditions

**Figure 4:**
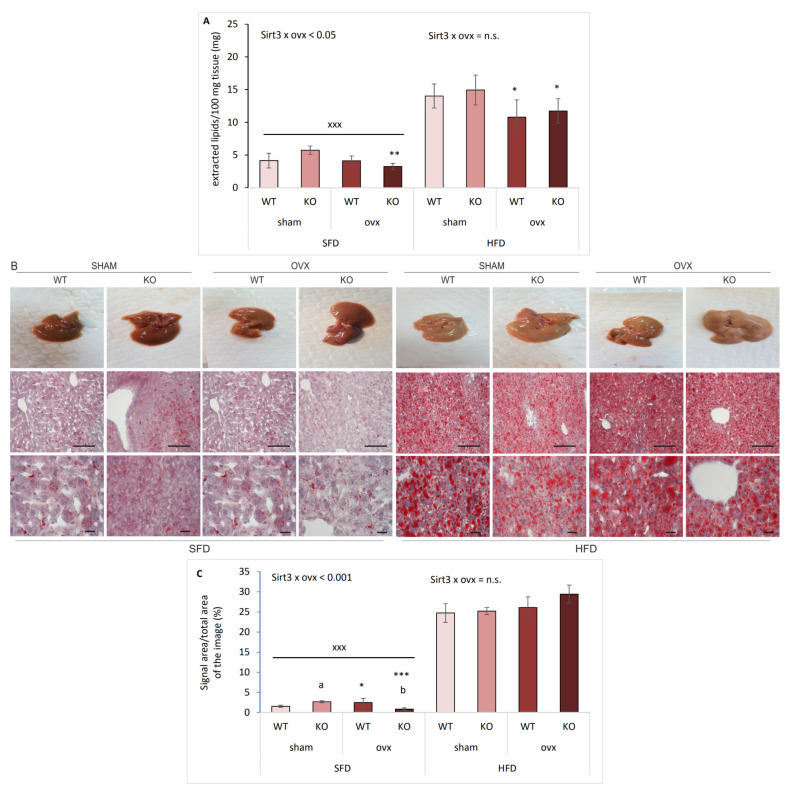
Sirt3 KO ovx mice have reduced lipid accumulation in SFD conditions. (**A**) Graphical display of hepatic total lipid content in sham and ovx Sirt3 WT and KO mice. SFD: KO ovx vs. sham (** *p* < 0.01). HFD: sham vs. ovx (* *p* < 0.05). HFD vs. SFD: ^xxx^ *p* < 0.001. (**B**) Representative macroscopic photographs of liver (upper panel) and histological analysis of liver sections with oil red staining (middle and lower panel). Scale bars: 100 μm (middle panel) and 20 μm (bottom panel). (**C**) Quantification of oil red signal in IHC samples using Image J. SFD: sham KO vs. WT (^a^ *p* < 0.05); WT sham vs. ovx (* *p* < 0.05); KO sham vs. ovx (*** *p* < 0.001); ovx WT vs. KO (^b^ *p* < 0.001). HFD: no change. SFD vs. HFD: ^xxx^ *p* < 0.001. Data are shown as mean ± SD. *n* = 4 mice per group.

To determine whether the increase in expression of genes involved in lipid metabolism was associated with hepatic lipid accumulation with respect to Sirt3 and ovx in SFD and HFD conditions, we measured lipid content using Folch extraction and performed the immunohistochemical (IHC) analysis of hepatic tissue using oil red staining. In SFD conditions, ovx reduced lipid content in Sirt3-depleted mice (** *p* < 0.01) (Figure 4A). Expectedly, HFD-fed mice had more lipid content than SFD-fed (^xxx^ *p* < 0.001), while ovx decreased lipid content in HFD conditions, irrespective of Sirt3 (* *p* < 0.05). IHC analysis showed interaction between Sirt3 and ovx in SFD-fed conditions: sham KO mice accumulated more lipids than WT (^a^ *p* < 0.01), and ovx WT mice accumulated more lipids than sham (* *p* < 0.05). Similar to Folch, oil red staining showed that SFD-fed mice depleted of both Sirt3 and ovary hormones accumulated less lipids compared to either ovx WT (^b^ *p* < 0.001) or sham KO mice (*** *p* < 0.001) (Figure 4B,C). In HFD conditions, all groups had higher lipid accumulation compared to their SFD-fed littermates (^xxx^ *p* < 0.001).

### 2.5. Sirt3 KO Ovx Mice Have Reduced Scd-1 Ratio and Less MUFA in SFD Conditions

**Figure 5:**
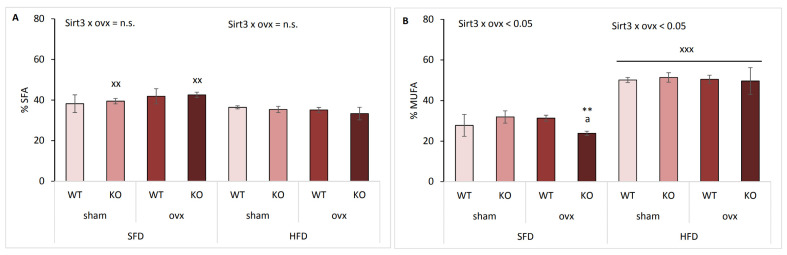

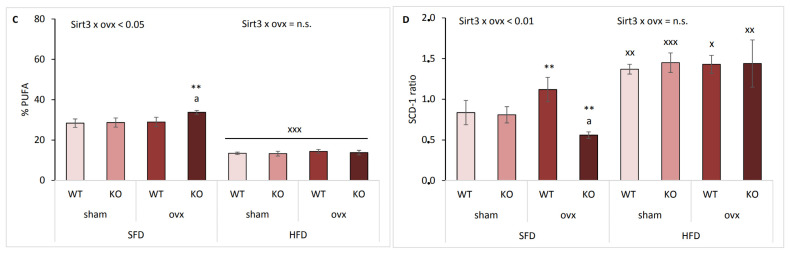
Sirt3 KO ovx mice have reduced Scd-1 ratio and MUFA in SFD conditions. Graphical display of hepatic fatty acid profile in sham and ovx Sirt3 WT and KO mice after 10 weeks of feeding with SFD or HFD. (**A**) % SFA. SFD: no changes. HFD: no changes. SFD vs. HFD: KO sham and ovx (^xx^ *p* < 0.01). (**B**) % MUFA. SFD: ovx WT vs. KO (^a^ *p* < 0.01); KO ovx vs. sham (** *p* < 0.01). HFD: no changes. SFD vs. HFD: ^xxx^ *p* < 0.001. (**C**) % PUFA: SFD: ovx WT vs. KO (^a^ *p* < 0.01); KO ovx vs. sham (**p < 0.01). HFD: no changes. SFD vs. HFD: ^xxx^ *p* < 0.001. (**D**) Scd-1 ratio. SFD: ovx WT vs. KO (^a^ *p* < 0.01); ovx vs. sham (** *p* < 0.01). HFD: no changes. SFD vs. HFD: KO sham (^xxx^ *p* < 0.001); WT sham and KO ovx (^xx^ *p* < 0.01); WT ovx (^x^ *p* < 0.05). Data are shown as mean ± SD. *n* = 4 mice per group.

To determine global changes in FAs composition, we determined total hepatic saturated FAs (SFA), monounsaturated FAs (MUFA), and polyunsaturated FAs (PUFA) by gas chromatography (GC). SFD-fed mice had a higher proportion of SFA compared to HFD-fed mice only in KO groups, irrespective of ovx (^xx^ *p* < 0.01) (Figure 5A). Proportions of MUFA were lowest in SFD-fed ovx KO mice, compared to both sham KO (** *p* < 0.01) and WT ovx mice (^a^ *p* < 0.01) (Figure 5B). HFD-fed mice had significantly higher proportions of MUFAs than SFD-fed mice (^xxx^ *p* < 0.001). The highest proportions of PUFAs were detected in SFD-fed ovx KO mice, compared to both sham KO (** *p* < 0.01) and WT ovx mice (^a^ *p* < 0.01) (Figure 5C). HFD-fed mice had lower PUFAs than SFD-fed mice (^xxx^ *p* < 0.001). These results indicate that in SFD-fed mice the depletion of Sirt3 and ovary hormones was associated with more hepatic PUFA than MUFA content and that HFD markedly shifted the dominant FAs in the liver from PUFAs to MUFAs following ten weeks of HFD feeding.

The most abundant SFAs were palmitate (C16:0), followed by stearate (C18:0) (Supplemental Figure S2A,B). SFD-fed ovx KO mice displayed accumulation of stearate compared to WT ovx mice (^a^ *p* < 0.001). Moreover, stearate was increased in all groups of SFD-fed mice compared to HFD-fed mice (^xxx^ *p* < 0.001) but did not influence the total SFA content in WT SFD mice compared to HFD, as observed in Figure 5A. Since mice depleted of both ovary hormones and Sirt3 on SFD had lower MUFA and higher PUFA levels, we wanted to explore which FAs contributed to the increase in PUFA to MUFA ratio. The main FAs in MUFA were palmitoleic, oleic, and vaccenic acid (Supplemental Figure S3A–C). Oleic acid, which is the main product of Scd-1 reaction and associates Scd-1 with the development of obesity and the metabolic syndrome [20], was significantly decreased in SFD-fed ovx KO mice (* *p* < 0.05), making all three main MUFAs reduced upon Sirt3 and ovary hormone deficiency. Furthermore, oleic acid levels were higher in all HFD-fed groups compared to SFD-fed groups (^xxx^ *p* < 0.001) which suggests that oleic acid is responsible for higher MUFA proportions after HFD feeding. The most abundant PUFA were linoleic acid (LNA), followed by arachidonic (AA) and docosahexaenoic acid (DHA) (Supplemental Figure S4A–C). Generally, lower total PUFAs in HFD-fed mice are the result of reduced levels of LNA, AA, and DHA.

Stearoyl-CoA desaturase-1 (Scd-1) plays the important role in lipogenesis and is expressed in metabolically active tissues, such as liver and adipose tissue [21]. Desaturation index (DI), the ratio of product to precursor FAs, is an indirect marker for tissue Scd-1 activity, which is decreased in conditions of inhibited Scd-1 activity [4]. In our study, DI was determined for palmitoleic/palmitic acid. In SFD conditions, Sirt3 depletion significantly attenuated (^a^ *p* < 0.001) ovx-mediated increase in Scd-1 ratio (** *p* < 0.01) (Figure 5D). HFD-fed mice displayed a similar Scd-1 ratio across all groups, which was significantly higher than SFD-fed groups (^x^ *p* < 0.05, ^xx^ *p* < 0.01, ^xxx^ *p* < 0.001). Higher Scd-1 ratio in HFD-fed mice that indicates higher Scd-1 activity may be due to higher MUFA content in HFD rather than direct product of Scd-1 activity.

### 2.6. Combination of Ovx and Sirt3 Depletion Increases Lipid Damage in SFD Conditions

**Figure 6:**
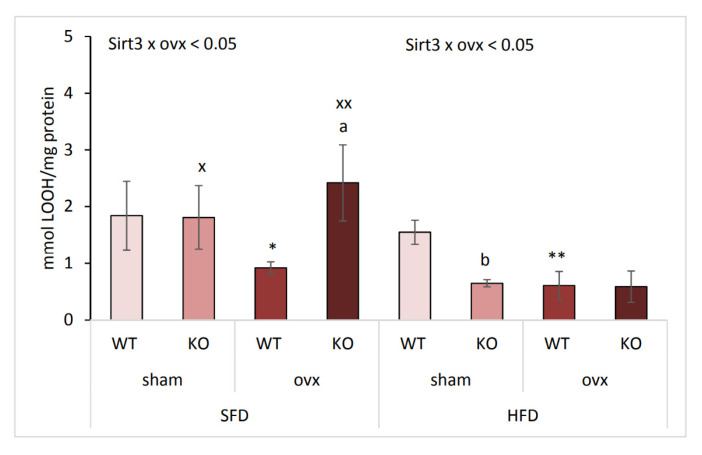
The combination of ovx and Sirt3 depletion increases lipid damage in SFD conditions. Graphical display of lipid hydroperoxide levels (LOOH) in sham and ovx Sirt3 WT and KO mice after 10 weeks of feeding with SFD or HFD. SFD: ovx WT vs. KO (^a^ *p* < 0.05); WT sham vs. ovx (* *p* < 0.05). HFD: sham KO vs. WT (^b^ *p* < 0.01); WT sham vs. ovx (** *p* < 0.01). SFD vs. HFD: KO sham (^x^ *p* < 0.05), KO ovx (^xx^ *p* < 0.01). Data are shown as mean ± SD. *n* = 4 mice per group.

Since ovx induces oxidative stress and Sirt3 ameliorates oxidative damage, we also determined by lipid hydroperoxide (LOOH) analysis the effect of ovx and Sirt3 depletion on oxidative damage to lipids with respect to type of diet. In SFD-fed sham mice no changes were observed in LOOH level, whereas upon ovx, KO mice displayed higher LOOH than WT mice (^a^ *p* < 0.01) indicating that combination of ovx and Sirt3 depletion resulted in increased lipid damage (Figure 6). Also, SFD-fed ovx WT mice had lower LOOH than sham WT (* *p* < 0.05). Within the HFD group, sham KO mice displayed lower LOOH than WT mice (^b^ *p* < 0.01). Ovx significantly reduced LOOH levels in WT mice (** *p* < 0.01), without the effect of Sirt3. SFD-fed KO mice had significantly higher lipid damage than HFD-fed KO mice, irrespective of ovx (^x^ *p* < 0.05, ^xx^ *p* < 0.01). Together, these data confirm that both Sirt3- and ovary hormone-depletion are associated with increased lipid damage only in SFD conditions.

### 2.7. Ovariectomized Females Maintain Mitochondrial CII-Driven Respiration in HFD Conditions

**Figure 7:**
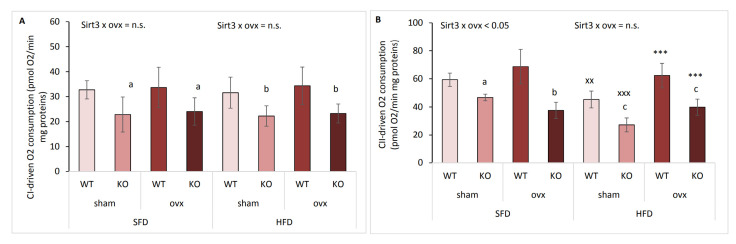
Ovariectomized females maintain mitochondrial CII-driven respiration in HFD conditions. Graphical displays of CI- and CII-driven mitochondrial respiration in sham and ovx Sirt3 WT and KO mice after 10 weeks of feeding with SFD or HFD. (**A**) CI-driven respiration. SFD: WT vs. KO (^a^ *p* < 0.01). HFD: WT vs. KO (^b^ *p* < 0.001). SFD vs. HFD: no changes. (**B**) CII-driven respiration. SFD: sham WT vs. KO (^a^ *p* < 0.01); ovx WT vs. KO (^b^ *p* < 0.001). HFD: WT vs. KO (^c^ *p* < 0.001); ovx vs. sham (*** *p* < 0.001). SFD vs. HFD: WT sham (^xx^ *p* < 0.01); KO sham (^xxx^ *p* < 0.001). Data are shown as mean ± SD. *n* = 4–6 mice per group.

To determine if mitochondrial function was affected by ovx and/or Sirt3 depletion, we measured CI-driven (malate + glutamate, ADP added) and CII-driven (succinate + rotenone, ADP added) active mitochondrial respiration by Clark-type electrode. In SFD conditions, KO mice exhibited lower CI-driven respiration than WT mice irrespective of ovx (^a^ *p* < 0.01) (Figure 7A). A similar effect was observed in HFD-fed mice (^b^ *p* < 0.001), indicating the importance of Sirt3 in active mitochondrial respiration. CII-driven respiration showed a similar trend as CI considering Sirt3 in SFD conditions, with KO mice having lower respiration than WT mice in both sham (^a^ *p* < 0.05) and to a greater extent in ovx group (^b^ *p* < 0.001) (Figure 7B). Following HFD, KO mice also showed lower CII-driven respiration than WT mice (^c^ *p* < 0.001). HFD generally decreased CII-driven respiration in both sham WT (^xx^ *p* < 0.01) and KO mice (^xxx^ *p* < 0.001) compared to their SFD-fed littermates. Surprisingly, CII-driven respiration was maintained in HFD-fed ovx mice, being higher than in HFD-fed sham mice (*** *p* < 0.001). These data indicate that mitochondrial CI-driven respiration depends only on Sirt3. Mitochondrial CII-driven respiration is also dependent on Sirt3, but is also diet and ovary hormone-dependent, with nutritional stress repressing CII- driven respiration only in sham mice.

### 2.8. Antioxidative Enzyme Activities Are Affected by Ovx and Type of Diet

**Figure 8:**
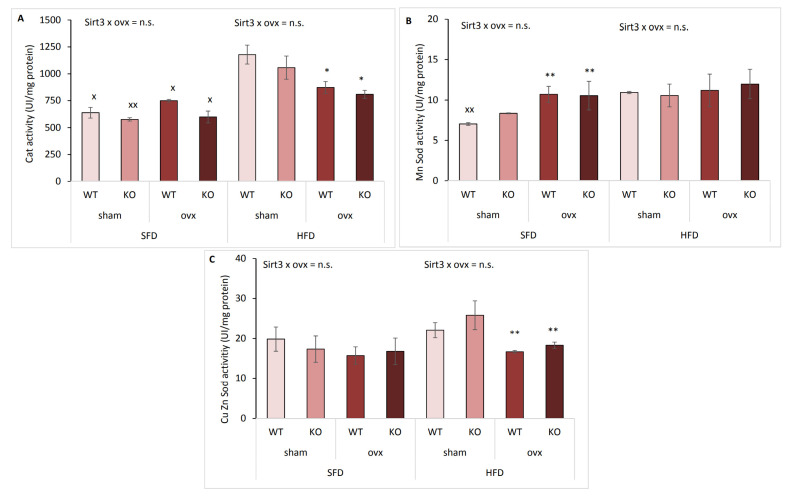
Antioxidative enzyme activities are affected by ovx and type of diet. Graphical display of antioxidant enzyme activities in sham and ovx Sirt3 WT and KO mice after 10 weeks of feeding with SFD or HFD. (**A**) Catalase activity. SFD: no changes. HFD: ovx vs. sham (* *p* < 0.05). SFD vs. HFD: WT sham, ovx WT and KO (^x^ *p* < 0.05), KO sham (^xx^ *p* < 0.01). (**B**) MnSod activity. SFD: ovx vs. sham (** *p* < 0.01). HFD: no changes. SFD vs. HFD: WT sham (^xx^ *p* < 0.01). (**C**) CuZnSod activity. SFD: no changes. HFD: ovx vs. sham (** *p* < 0.01). SFD vs. HFD: no changes. Data are shown as mean ± SD. *n* = 4 mice per group.

Since we previously found that the antioxidative enzyme system was affected in a sex-related manner with respect to the type of diet [13], we analyzed the activities of major antioxidant enzymes: catalase (Cat), manganese superoxide dismutase (MnSod), and copper-zinc superoxide dismutase (CuZnSod). Expectedly, Cat activity was unchanged within SFD-fed mice (Figure 8A). Within HFD, ovx mice had lower Cat activity than sham mice irrespective of Sirt3 (* *p* < 0.05), but it was still significantly increased compared to SFD-fed groups, indicating increased activity of Cat following HFD in all groups (^x^ *p* < 0.05, ^xx^ *p* < 0.01). Interestingly, in the case of MnSod activity, hormone depletion increased it in SFD conditions (** *p* < 0.01) regardless of Sirt3 (Figure 8B). HFD-fed mice had no change in MnSod activity between groups, and only WT sham mice had increased activity compared to their SFD-fed littermates (^xx^ *p* < 0.01). Similar to Cat activity, CuZnSod activity remained unchanged within SFD-fed mice, but also decreased in ovx mice compared to sham mice (** *p* < 0.01) following HFD (Figure 8C). This indicates that hormone depletion decreases both Cat and CuZnSod activity in HFD-fed mice. Overall, the activities of antioxidant enzymes were not affected by Sirt3, only by ovx and type of diet.

## 3. Discussion

Obesity and metabolic syndrome represent major health problems worldwide [22], development of which is associated with metabolic and hormonal changes occurring during the lifespan in both sexes. Since diet can additionally favor the disease progression, understanding these disorders and their causes in both sexes has one of the highest priorities. Although many studies showed that females have better protection against HFD-induced metabolic stress than males [23,24,25,26], the combined effect of main mitochondrial deacetylase Sirt3 and ovary hormones in regulating metabolic stress in vivo has not yet been investigated. To test our hypothesis that females’ protection from HFD was attributed to the synergistic effect of female sex hormones and Sirt3, we investigated the effects of Sirt3 depletion and ovarian hormone deficiency (ovx) on metabolic parameters, mitochondrial function, antioxidant system, and lipid profile in the liver of SFD- and HFD-fed female 129S mice.

Our results indicate that in SFD-fed Sirt3 WT mice, ovx resulted in a following compensatory response to stress: increased *pgc1-α* and its downstream target Sirt3, accompanied with maintained mitochondrial function, increased FAs synthesis (higher Scd-1 activity [27]), and MnSod activity coupled with lower levels of LOOH. Ovx also induced *pparα* expression, known for upregulation of hepatic lipid metabolism proteins [28]. Although it has been reported that Sirt3 downregulates Scd-1 in male mice [29], here the ovx in females caused upregulation of Sirt3 and increased Scd-1. However, ovary hormone and Sirt3 depletion caused downregulation of Scd-1, indicating that Sirt3 participates in the regulation of Scd-1 in the absence of ovary hormones. It could be possible that Sirt3 increases Scd-1 by deacetylation and inhibition of its negative regulator STAT3 [21], however, this needs to be confirmed in future studies. We suggest that, although in SFD conditions ovx mice have increased FA synthesis, the upregulation of Sirt3 compensates for the loss of ovary hormones by maintaining mitochondrial metabolism and preventing oxidative damage. Consistent with previous reports indicating that increased Sirt3 expression is in association with decreased oxidative stress [30,31], these results show that in SFD conditions Sirt3 protects from mitochondrial dysfunction and oxidative damage in ovx females.

Previous studies showed that the genetic background of mice strain plays a significant role in the progression of disease under the same conditions of HFD. Although 129S mice develop features of metabolic syndrome with lower severity compared to some other strains [32,33,34,35,36], 129S Sirt3 KO mice are extremely useful in studying the role of FA oxidation in diabetes, steatosis, and life span [4,37]. However, the conducted metabolism-associated studies involved male mice only. Although our strain of mice develops obesity with lower severity, weight gain was significantly affected in KO ovx mice: these females were most resistant towards gaining weight on SFD but most sensitive upon HFD. Decreased body weight gain in SFD conditions was associated with reduced *pparα* expression, lower Scd-1 activity, and lowest hepatic lipid content. The inhibition of Scd-1 is found to shift FA metabolism towards increased FA oxidation pathway [38], which participates in alleviation of obesity by burning off excessive accumulated lipids [39]. Thus, this may explain their resistance towards gaining weight under SFD conditions. Other parameters that indicate reduced Scd-1 activity are lowered content of oleic in favor of stearic acid, and elevated PUFAs, known as powerful inhibitors of Scd-1 gene expression, specifically AA and DHA (reviewed in [21]). Also, ovx KO females have more compromised CII-driven mitochondrial respiration associated with the highest lipid oxidative damage. In conclusion, we propose that combined loss of both Sirt3 and ovary hormones in SFD conditions results in the lowest body weight gain as a consequence of reduced *pparα* expression and Scd-1 activity, compromised CII-driven respiration, and higher lipid oxidative damage, thus pointing towards unquestionable protective effect of combined Sirt3 and ovary hormones in maintaining metabolic and oxidative homeostasis in female mice.

HFD-fed ovx mice usually exhibit an obese phenotype with decreased energy expenditure, referring to the importance of the estrogen signaling pathway in the maintenance of energy homeostasis [40]. However, the role of Sirt3 in these processes is not clear. In agreement with previous data regarding the protective role of Sirt3 and ovary hormones in obesity [11,41], HFD-fed ovx KO females displayed the highest body weight gain, but interestingly, lower lipid accumulation than sham mice. This is consistent with our previous study if we compare ovx KO females to male KO mice, which also showed to have the lowest lipid accumulation on HFD, indicating their increased reliance on FAs which probably compensated for their impaired glucose uptake [13]. At the time, we assumed that the observed sex-related differences in lipid accumulation are present only in Sirt3 KO mice, but now we demonstrate the same effect in both ovx WT and KO females, suggesting the important role of ovary hormones in these processes. In addition, HFD-fed ovx mice maintained their CII-driven respiration, possibly due to increased MUFAs (palmitoleic and vaccenic acids) known for enhancing FA oxidation, i.e., energy consumption by raising mitochondrial respiratory complexes and ATP production [42]. Whilst mitochondria are the primary site of β-oxidation for energy production in the form of ATP, peroxisomal β-oxidation is involved in biosynthesis pathways with the end product acetyl CoA and H_2_O_2_ [43]. Based on observed lower mitochondrial CII-driven respiration in HFD-fed sham mice, as well as higher Cat, a common peroxisomal ROS scavenging enzyme, we propose that these females depend more on peroxisomal FA oxidation. This indicates that HFD aggravates their metabolic parameters by compromising mitochondrial function and activating ROS-induced upregulation of antioxidant enzymes.

Although ovx females on HFD have lower lipid accumulation, depletion of either ovary hormones or both ovary hormones and Sirt3 changes the expression of *cyp2e1* and *cyp4a14*. Higher expression of either of these genes indicates that these mice are prone to NAFLD since it is known that both of them induce hepatosteatosis, with an increase in ROS and oxidative stress [18,44]. In addition, the expression level of the *ho-1* gene, which has been reported to attenuate oxidative stress and prevent nonalcoholic steatohepatitis (NASH) [45], is upregulated in WT and decreased in KO ovx mice. Thus, in ovx females, Sirt3 may protect from NASH by upregulation of *ho-1*, while in the absence of Sirt3 the expression of *ho-1* is abolished, making these mice more susceptible to the development of NASH caused by HFD. From the two analyzed *cyp* genes, *cyp4a14* showed to be more involved in the induction of NAFLD [46] and *cyp2e1* in alcoholic hepatitis [47]. This may be the reason that *cyp4a14* has higher expression in all HFD groups, and highest in KO ovx groups in either SFD or HFD conditions. Overall, the expression of these genes indicates that, despite lower lipid accumulation in ovx females which may be due to differential dependence on peroxisomal and mitochondrial β-oxidation of FA, mice lacking ovary hormones and especially in combination with Sirt3 depletion are more prone to NAFLD.

The limitation of the study is that parameters such as Scd-1 which are derived from the ratio of particular FAs may not be fully reliable indicators of Scd-1 activity in HFD conditions because their levels highly depend on fat composition in the diet. Several authors stated that lipogenesis was reduced in mouse models of HFD feeding (reviewed in [48]). In addition, in this study, we included only young adult females, and ovx might not have such adverse effects as it would have in older females. Further studies in senescent mice are needed to compare parameters involved in lipid metabolism, mitochondrial function, and antioxidant system, which we plan to conduct in near future.

Despite these limitations, this research adds to our previous studies and confirms our hypothesis that protection against harmful effects of HFD in female mice is attributed to the combined effect of female sex hormones and Sirt3. With this, we add to the knowledge on the prevention of metabolic dysfunction, thus contributing to preclinical research and supporting future studies for the development of sex-related therapeutic agents for metabolic syndrome and associated diseases.

## 4. Materials and Methods

### 4.1. Animal Model and Experimental Design

129S1/SvImJ WT (Stock No: 002448) and Sirt3 KO (Stock No: 012755, Jackson Laboratory, Bar Harbor, ME, USA) female mice were housed in standard conditions (three females per cage, 22 °C, 50–70% humidity, 12 h light / 12 h darkness cycle). Ovariectomy (ovx) and sham surgery were performed at 7 weeks of age under ketamine/xylazine anesthesia (Ketamidor 10%, Richter pharma Ag, Wels, Austria; Xylazine 2%, Alfasan International, Woerden, Netherlands). Since low levels of E2 are normally detected in ovariectomized females due to other endogenous E2 sources (reviewed in [49]), plasma E2 levels were not used as an indicator of the efficiency of ovx. Instead, the success of ovx was checked by analyzing vaginal smear during five consecutive days after the surgery [50]. After recovery, mice were placed on either a standard fat diet (SFD, 11.4% fat, 62.8% carbohydrates, 25.8% proteins; Mucedola, Settimo Milanese, Italy) or a high fat diet (HFD, 58% fat, 24% carbohydrates, 18% proteins; Mucedola, Settimo Milanese, Italy) for 10 weeks. Body weight was measured once a week, as well as glucose level (glucometer StatStrip Xpress-I, Nova Biomedical, GmbH, Mörfelden-Walldorf, Germany) after 6 h of fasting, in a blood drop from the tail vein. After 10 weeks of feeding, mice were sacrificed and liver was used either fresh or was stored in liquid nitrogen or at −80°C, depending on the analysis. Animal experiments were done within the project funded by the Croatian Science Foundation, project ID: IP-014-09-4533, approved on 01/09/2015. All procedures were approved by the Ministry of Agriculture of Croatia (No: UP/I-322-01/15-01/25 525-10/0255-15-2 from 20th July 2015) and carried out following the EU Directive 2010/63/EU-associated guidelines.

### 4.2. Histology and Oil Red O Staining

A histological analysis of samples taken from the right liver lobe for all experimental groups was performed as described previously [13]. Fat vacuoles in hepatocytes of frozen sections were visualized by Oil Red O dye (Sigma Aldrich, St. Louis, MO, USA) according to the previous protocol [13], with the following modifications: Oil Red O dye was prepared in isopropanol (0.5% Oil Red O solution), the sections (8 µm) of tissues embedded in Optimal Cutting Temperature medium (O.C.T 4583, Sakura Finetek, Torrance, CA, USA) were air-dried for 1 h and the tissue was fixed in ice-cold 10% formalin for 5 min, washed with dH_2_O, and conditioned for staining by brief dipping of slides in 60% isopropanol. The tissue sections were stained with Oil Red O dye in the dark, at room temperature for 15 min, and then washed by rinsing in 60% isopropanol, and incubated for 5 min in dH_2_O, followed by staining with Mayer’s hematoxylin (Dako, Histological staining reagent S3309, Santa Clara, CA, USA) for 1 min and washing with tap water and dH_2_O, and were finally mounted in aqueous mounting medium (Dako Faramount Aqueous Mounting Medium S3025, Agilent Technologies, Santa Clara, CA, USA). An analysis of the stained liver sections was done using an Olympus BX51 microscope (Tokyo, Japan) with associated software analysis.

### 4.3. Total Lipid Extraction and GC Lipid Analysis

Liver samples were snap-frozen and stored at −80 °C until analysis. Total lipids were extracted from liver tissue according to a modified Folch procedure as described previously [13,51]. The lipid extract was treated with 0.5 M KOH/MeOH for 20 min at room temperature, and the corresponding FA methyl esters (FAMEs) were formed and analyzed by gas chromatography (GC). GC analyses of total FAs were performed by Varian 450-GC equipped with a flame ionization detector (Varian Medical Systems, Houten, Netherlands). A Stabilwax column (crossbond carbowax polyethylene glycol, 60 m × 0.25 mm) was used as a stationary phase at a programmed temperature with helium as the carrier gas. The heating was carried out at a temperature of 150 °C for 1 min followed by an increase of 1 °C/min up to 250 °C. Methyl esters were identified by comparison with the retention times of commercially available standard mixtures (Marine oil FAME mix, Restek Corporation, Bellefonte, PA, USA).

### 4.4. Lipid Hydroperoxide Analysis

Liver samples were snap-frozen and stored at −80 °C until analysis. Lipids were extracted according to the modified method previously described [52]. Briefly, 0.1 g of liver tissue was cut and homogenized in PBS. Lipid extraction started by adding 2.5 mL of CHCl_3_ to the homogenate, followed by vigorous shaking. The solution was washed with 0.75 mL of 0.034% MgCl_2_ and centrifuged (2 min, 3000× *g*) and the aqueous layer was drawn off by aspiration using a Pasteur pipette. The washing procedure was repeated with 1.25 mL of 2 M KCl/MeOH (4:1, v/v) to eliminate all proteins and non-lipid contaminants. CHCl_3_ layer was finally washed with CHCl_3_/MeOH (2:1, v/v) and centrifuged (5 min, 3000× *g*). The organic layer containing lipids was carefully transferred to a glass tube and solvent was removed on a rotary evaporator. After weighing, the lipids were stored at −20 °C until the analysis of lipid hydroperoxides (LOOH). Spectrophotometric ferric thiocyanate assay was used for the determination of LOOH concentration. The analyzed samples were prepared by diluting with a deaerated mixture of CH_2_Cl_2_ /MeOH (2:1, v/v). The concentrations of LOOH were calculated by using the molar absorptivity of the complex [FeNCS]^2 +^ formed per mol of LOOH, 58 440 dm^3^ mol^−1^ cm^−1^, at 500 nm [53].

### 4.5. RNA Isolation and Quantitative Real-Time PCR Analysis

Liver samples were snap-frozen and stored in liquid nitrogen until analysis. Total RNA from liver samples was isolated using TRIzol reagent (Invitrogen, Waltham, MA, USA). RNA was treated with DNAse (TURBO DNA-free Kit, Thermo Fisher Scientific, Waltham, MA, USA) followed by reverse transcription using a High-Capacity cDNA Reverse Transcription Kit (Thermo Fisher Scientific, Waltham, MA, USA). For real-time PCR analysis, an ABI 7300 sequence detection system (Foster City, CA, USA) was used. To quantify the relative mRNA expression of *cyp2e1*, *cyp4a14*, *pparα*, *pgc1-α*, and *ho-1* (Supplementary Table S1) the comparative CT (^ΔΔ^CT) method according to the Taqman^®^ Gene Expression Assays Protocol (Applied Biosystems, Foster City, CA, USA) was used. The data on the graphs are shown as the fold-change in gene expression, which is normalized to the endogenous reference gene (*β-actin*) and relative to SFD-fed sham WT females.

### 4.6. Protein Isolation and Western Blot Analysis

Liver samples were snap-frozen and stored in liquid nitrogen until analysis. Liver proteins were prepared in Ripa buffer (supplemented with cOmplete™, EDTA-free Protease Inhibitor Cocktail tablets (Roche, Basel, Switzerland)) using an ice-jacketed Potter-Elvehjem homogenizer (1300 rpm; Thomas Scientific, Swedesboro, NJ, USA) according to our standard protocol [13]. Proteins (15 μg/μL) were resolved by SDS-PAGE and transferred onto a PVDF membrane (Roche, Basel, Switzerland). Membranes were blocked and incubated with primary antibodies (Supplementary Table S2) overnight at 4°C. For chemiluminescence detection, an appropriate horseradish peroxidase (HRP)-conjugated secondary antibody was used. AmidoBlack (Sigma Aldrich, St. Louis, MO, USA) was used for total protein normalization. The Alliance 4.7 Imaging System (UVITEC, Cambridge, UK) was used for the detection of immunoblots using an enhanced chemiluminescence kit (Thermo Fischer Scientific, Waltham, MA, USA).

### 4.7. Analysis of Antioxidative Enzyme Activities

Liver samples were snap-frozen and stored in liquid nitrogen until analysis. Antioxidative enzyme activities were analyzed in liver homogenates prepared in PBS supplemented with cOmplete™, EDTA-free Protease Inhibitor Cocktail tablets (Roche, Basel, Switzerland) using an ice-jacketed Potter-Elvehjem homogenizer (1300 rpm; Thomas Scientific, Swedesboro, NJ, USA). Superoxide dismutase (Sod) activities were determined using a Ransod kit (Randox Laboratories, Crumlin, UK) according to the manufacturer’s recommendations. The catalase (Cat) activity was done as previously described [54], by measuring the change in absorbance (at 240 nm) in the reaction mixture (10 mM H_2_O_2_ and 50 mM PBS (pH 7.0)) during the interval of 30 s following sample addition.

### 4.8. Mitochondria Isolation and Oxygen Consumption

Mice liver mitochondria were isolated from fresh liver by differential centrifugation as described previously [13]. Isolated mitochondria were kept in the isolation buffer (250 mM sucrose, 2 mM EGTA, 0.5% fatty acid-free BSA, 20 mM Tris-HCl, pH 7.4) until the experiment on the Clark-type electrode (Oxygraph, Hansatech Instruments Ltd., Pentney, UK) in an airtight 1.5 mL chamber at 35°C. Mitochondria (800 μg protein) were resuspended in a 500 μL respiration buffer (200 mM sucrose, 20 mM Tris-HCl, 50 mM KCl, 1 mM MgCl_2_·6H_2_O, 5 mM KH_2_PO_4_, pH 7,0) for the determination of oxygen consumption. Complex I assessment samples were incubated with 2.5 mM glutamate and 1.25 mM malate. Complex II assessment samples were incubated with 2 μM rotenone and 10 mM succinate. Mitochondrial respiration was accelerated by the addition of ADP (2 mM final concentration) for state 3 respiration measurements. Then, ATP synthesis was terminated by adding oligomycin (6.25 nM final concentration) to achieve state 4 rate. To inhibit mitochondrial respiration, 2 μM antimycin A was used. Oxygen consumption is calculated in nmol/min/mg protein.

### 4.9. Statistical Analysis

For the statistical analysis of data, SPSS for Windows (v.17.0, IBM, Armonk, NY, USA) was used. A Shapiro–Wilk test was used before all analyses to test the samples for normality of distribution. Since all data followed a normal distribution, parametric tests for multiple comparisons were performed: an unpaired Student’s t-test for comparisons between SFD and HFD, and a two-way ANOVA for the interaction effect of Sirt3 and ovx within each diet. If a significant interaction was observed, all pairwise comparisons were made between groups, using Tukey’s post-hoc test with Bonferroni’s correction. Significance was set at *p* < 0.05. On graphical displays, the indicator of the differences between SFD and HFD was marked as x; the indicator of differences between WT and KO (the effect of Sirt3) was marked as a letter (a, b, etc.); the indicator of differences between sham and ovx (the effect of ovx) was marked as *.

## Data Availability

The data presented in this study are available on request from the corresponding author.

## Supplementary Materials

The following are available online at https://www.mdpi.com/article/10.3390/ijms22084277/s1, Figure S1: Observations of uterus deterioration and vaginal smears of control (sham) and ovariectomized (ovx) mice. Figure S2: Graphical display of hepatic SFA content in sham and ovx Sirt3 WT and KO mice after 10 weeks of feeding with SFD or HFD. Figure S3: Graphical display of hepatic MUFA content in sham and ovx Sirt3 WT and KO mice after 10 weeks of feeding with SFD or HFD. Figure S4: Graphical display of hepatic PUFA content in sham and ovx Sirt3 WT and KO mice after 10 weeks of feeding with SFD or HFD. Table S1: Assays (Taqman^®^ Applied Biosystems, UK) used for the real time quantitative PCR. Table S2: Antibodies used in this study for the Western blot analyses.

## References

[1] Bonomini F., Rodella L.F., Rezzani R. (2015). Metabolic syndrome, aging and involvement of oxidative stress. Aging Dis..

[2] Rui L. (2014). Energy metabolism in the liver. Compr. Physiol..

[3] Kakimoto P.A., Kowaltowski A.J. (2016). Effects of high fat diets on rodent liver bioenergetics and oxidative imbalance. Redox Biol..

[4] Hirschey M.D., Shimazu T., Jing E., Grueter C.A., Collins A.M., Aouizerat B., Stančáková A., Goetzman E., Lam M.M., Schwer B. (2011). SIRT3 deficiency and mitochondrial protein hyperacetylation accelerate the development of the metabolic syndrome. Mol. Cell.

[5] Haas J.T., Francque S., Staels B. (2016). Pathophysiology and mechanisms of nonalcoholic fatty liver disease. Annu. Rev. Physiol..

[6] Austad S.N. (2006). Why women live longer than men: Sex differences in longevity. Gend. Med..

[7] Kruger D.J., Nesse R.M. (2006). An evolutionary life-history framework for understanding sex differences in human mortality rates. Hum. Nat..

[8] Mauvais-Jarvis F., Bairey Merz N., Barnes P.J., Brinton R.D., Carrero J.J., DeMeo D.L., De Vries G.J., Epperson C.N., Govindan R., Klein S.L. (2020). Sex and gender: Modifiers of health, disease, and medicine. Lancet.

[9] Ko S.H., Kim H.S. (2020). Menopause-associated lipid metabolic disorders and foods beneficial for postmenopausal women. Nutrients.

[10] Palmisano B.T., Zhu L., Stafford J.M. (2017). Role of estrogens in the regulation of liver lipid metabolism. Adv. Exp. Med. Biol..

[11] Zhang J., Xiang H., Liu J., Chen Y., He R.R., Liu B. (2020). Mitochondrial Sirtuin 3: New emerging biological function and therapeutic target. Theranostics.

[12] Lombard D.B., Alt F.W., Cheng H.L., Bunkenborg J., Streeper R.S., Mostoslavsky R., Kim J., Yancopoulos G., Valenzuela D., Murphy A. (2007). Mammalian Sir2 homolog SIRT3 regulates global mitochondrial lysine acetylation. Mol. Cell Biol..

[13] Pinteric M., Podgorski I.I., Hadzija M.P., Bujak I.T., Dekanic A., Bagaric R., Farkas V., Sobocanec S., Balog T. (2020). Role of sirt3 in differential sex-related responses to a high-fat diet in mice. Antioxidants.

[14] Simpkins J.W., Yi K.D., Yang S.H., Dykens J.A. (2010). Mitochondrial mechanisms of estrogen neuroprotection. Biochim. Biophys. Acta Gen. Subj..

[15] Gruber C.J., Tschugguel W., Schneeberger C., Huber J.C. (2002). Production and Actions of Estrogens. N. Engl. J. Med..

[16] Liang H., Ward W.F. (2006). PGC-1α: A key regulator of energy metabolism. Adv. Physiol. Educ..

[17] Brandt J.M., Djouadi F., Kelly D.P. (1998). Fatty acids activate transcription of the muscle carnitine palmitoyltransferase I gene in cardiac myocytes via the peroxisome proliferator-activated receptor α. J. Biol. Chem..

[18] Leclercq I.A., Farrell G.C., Field J., Bell D.R., Gonzalez F.J., Robertson G.R. (2000). CYP2E1 and CYP4A as microsomal catalysts of lipid peroxides in murine nonalcoholic steatohepatitis. J. Clin. Investig..

[19] Chung H.T., Ryter S.W., Kim H.P. (2013). Heme oxygenase-1 as a novel metabolic player. Oxid. Med. Cell. Longev..

[20] Jeffcoat R. (2007). Obesity—A perspective based on the biochemical interrelationship of lipids and carbohydrates. Med. Hypotheses.

[21] Mauvoisin D., Mounier C. (2011). Hormonal and nutritional regulation of SCD1 gene expression. Biochimie.

[22] Saklayen M.G. (2018). The global epidemic of the metabolic syndrome. Curr. Hypertens. Rep..

[23] Hwang L.L., Wang C.H., Li T.L., Chang S.D., Lin L.C., Chen C.P., Chen C.T., Liang K.C., Ho I.K., Yang W.S. (2010). Sex differences in high-fat diet-induced obesity, metabolic alterations and learning, and synaptic plasticity deficits in mice. Obesity.

[24] Dorfman M.D., Krull J.E., Douglass J.D., Fasnacht R., Lara-Lince F., Meek T.H., Shi X., Damian V., Nguyen H.T., Matsen M.E. (2017). Sex differences in microglial CX3CR1 signalling determine obesity susceptibility in mice. Nat. Commun..

[25] Yang Y., Smith D.L., Keating K.D., Allison D.B., Nagy T.R. (2014). Variations in body weight, food intake and body composition after long-term high-fat diet feeding in C57BL/6J mice. Obesity.

[26] Lainez N.M., Jonak C.R., Nair M.G., Ethell I.M., Wilson E.H., Carson M.J., Coss D. (2018). Diet-induced obesity elicits macrophage infiltration and reduction in spine density in the hypothalami of male but not female mice. Front. Immunol..

[27] Miller C.W., Ntambi J.M. (1996). Peroxisome proliferators induce mouse liver stearoyl-CoA desaturase 1 gene expression. Proc. Natl. Acad. Sci. USA.

[28] Kersten S. (2014). Integrated physiology and systems biology of PPARα. Mol. Metab..

[29] Zhang T., Liu J., Shen S., Tong Q., Ma X., Lin L. (2020). SIRT3 promotes lipophagy and chaperon-mediated autophagy to protect hepatocytes against lipotoxicity. Cell Death Differ..

[30] Liu J., Li D., Zhang T., Tong Q., Ye R.D., Lin L. (2017). SIRT3 protects hepatocytes from oxidative injury by enhancing ROS scavenging and mitochondrial integrity. Cell Death Dis..

[31] Zheng J., Shi L., Liang F., Xu W., Li T., Gao L., Sun Z., Yu J., Zhang J. (2018). Sirt3 ameliorates oxidative stress and mitochondrial dysfunction after intracerebral hemorrhage in diabetic rats. Front. Neurosci..

[32] Biddinger S.B., Almind K., Miyazaki M., Kokkotou E., Ntambi J.M., Kahn C.R. (2005). Effects of diet and genetic background on sterol regulatory element-binding protein-1c, stearoyl-CoA desaturase 1, and the development of the metabolic syndrome. Diabetes.

[33] Almind K., Kahn C.R. (2004). Genetic determinants of energy expenditure and insulin resistance in diet-induced obesity in mice. Diabetes.

[34] Ussar S., Griffin N.W., Bezy O., Fujisaka S., Vienberg S., Softic S., Deng L., Bry L., Gordon J.I., Kahn C.R. (2015). Interactions between gut microbiota, host genetics and diet modulate the predisposition to obesity and metabolic syndrome. Cell Metab..

[35] Asgharpour A., Cazanave S.C., Pacana T., Seneshaw M., Vincent R., Banini B.A., Kumar D.P., Daita K., Min H.K., Mirshahi F. (2016). A diet-induced animal model of non-alcoholic fatty liver disease and hepatocellular cancer. J. Hepatol..

[36] Sabidó E., Wu Y., Bautista L., Porstmann T., Chang C.Y., Vitek O., Stoffel M., Aebersold R. (2013). Targeted proteomics reveals strain-specific changes in the mouse insulin and central metabolic pathways after a sustained high-fat diet. Mol. Syst. Biol..

[37] Hirschey M.D., Shimazu T., Goetzman E., Jing E., Schwer B., Lombard D.B., Grueter C.A., Harris C., Biddinger S., Ilkayeva O.R. (2010). SIRT3 regulates mitochondrial fatty-acid oxidation by reversible enzyme deacetylation. Nature.

[38] Flowers M.T., Ntambi J.M. (2008). Role of stearoyl-coenzyme A desaturase in regulating lipid metabolism. Curr. Opin. Lipidol..

[39] Vijayakumar R.S., Lin Y., Shia K.-S., Yeh Y.-N., Hsieh W.-P., Hsiao W.-C., Chang C.-P., Chao Y.-S., Hung M.-S. (2012). Induction of fatty acid oxidation resists weight gain, ameliorates hepatic steatosis and reduces cardiometabolic risk factors. Int. J. Obes..

[40] Heine P.A., Taylor J.A., Iwamoto G.A., Lubahn D.B., Cooke P.S. (2000). Increased adipose tissue in male and female estrogen receptor-α knockout mice. Proc. Natl. Acad. Sci. USA.

[41] Leeners B., Geary N., Tobler P.N., Asarian L. (2017). Ovarian hormones and obesity. Hum. Reprod. Update.

[42] Cruz M.M., Lopes A.B., Crisma A.R., de Sá R.C.C., Kuwabara W.M.T., Curi R., de Andrade P.B.M., Alonso-Vale M.I.C. (2018). Palmitoleic acid (16:1n7) increases oxygen consumption, fatty acid oxidation and ATP content in white adipocytes. Lipids Health Dis..

[43] Demarquoy J., Le Borgne F. (2015). Crosstalk between mitochondria and peroxisomes. World J. Biol. Chem..

[44] Abdelmegeed M.A., Banerjee A., Yoo S.H., Jang S., Gonzalez F.J., Song B.J. (2012). Critical role of cytochrome P450 2E1 (CYP2E1) in the development of high fat-induced non-alcoholic steatohepatitis. J. Hepatol..

[45] Nan Y., Wang R., Zhao S., Han F., Wu W.J., Kong L., Fu N., Kong L., Yu J. (2010). Heme oxygenase-1 prevents non-alcoholic steatohepatitis through suppressing hepatocyte apoptosis in mice. Lipids Health Dis..

[46] Zhang X., Li S., Zhou Y., Su W., Ruan X., Wang B., Zheng F., Warner M., Gustafsson J.Å., Guan Y. (2017). Ablation of cytochrome P450 omega-hydroxylase 4A14 gene attenuates hepatic steatosis and fibrosis. Proc. Natl. Acad. Sci. USA.

[47] Leung T.M., Nieto N. (2013). CYP2E1 and oxidant stress in alcoholic and non-alcoholic fatty liver disease. J. Hepatol..

[48] Duarte J.A.G., Carvalho F., Pearson M., Horton J.D., Browning J.D., Jones J.G., Burgess S.C. (2014). A high-fat diet suppresses de novo lipogenesis and desaturation but not elongation and triglyceride synthesis in mice. J. Lipid Res..

[49] Cui J., Shen Y., Li R. (2013). Estrogen synthesis and signaling pathways during aging: From periphery to brain. Trends Mol. Med..

[50] Mačak Šafranko Ž., Sobočanec S., Šarić A., Jajčanin-Jozić N., Krsnik Ž., Aralica G., Balog T., Abramić M., Šafranko Ž.M., Sobočanec S. (2015). The effect of 17β-estradiol on the expression of dipeptidyl peptidase III and heme oxygenase 1 in liver of CBA/H mice. J. Endocrinol. Invest..

[51] Ways P., Hanahan D.J. (1964). Characterization and quantification of red cell lipids in normal man. J. Lipid Res..

[52] Bujak M., Bujak I.T., Sobočanec S., Mihalj M., Novak S., Cosić A., Levak M.T., Kopačin V., Mihaljević B., Balog T. (2018). Trefoil Factor 3 Deficiency Affects Liver Lipid Metabolism. Cell. Physiol. Biochem..

[53] Mihaljević B., Katušin-Ražem B., Ražem D. (1996). The reevaluation of the ferric thiocyanate assay for lipid hydroperoxides with special considerations of the mechanistic aspects of the response. Free Radic. Biol. Med..

[54] Aebi H. (1984). Catalase in vitro. Methods Enzymol..

