# Supplementary material for: Chronic High Fat Diet Intake Impairs Hepatic Metabolic Parameters in Ovariectomized Sirt3 KO Mice
Source: Int J Mol Sci. 2021 Apr 20;22(8):4277. doi: 10.3390/ijms22084277 (PMC8074326; doi:10.3390/ijms22084277)
Supplement: Supplementary file 1 [file ijms-22-04277-s001.zip › ijms-1177635-supplementary.pdf]

Supplementary material

Chronic High Fat Diet Intake Impairs Hepatic Metabolic Parameters in Ovariectomized Sirt3 KO Mice

Marija Pinterić [1†], Iva I. Podgorski [1†], Marijana Popović Hadžija [1], Ivana Tartaro Bujak [2], Ana Tadijan [1], Tihomir Balog [1], Sandra Sobočanec [1*]

[1] Division of Molecular Medicine, Ruđer Bošković Institute, 10000 Zagreb, Croatia

[2] Division of Materials Chemistry, Ruđer Bošković Institute, 10000 Zagreb, Croatia

[†] Equal contribution

*****Correspondence: ssoboc@irb.hr; tel.+385-1-4561-172

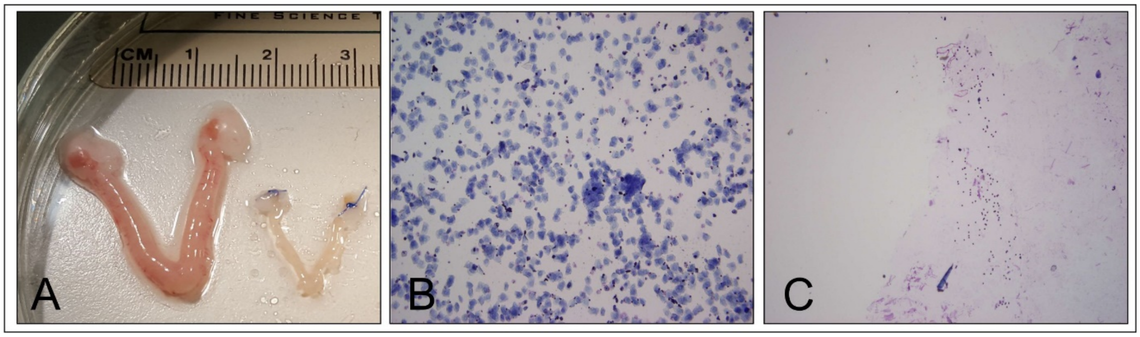

Figure S1. *Observations of uterus deterioration and vaginal smears of control (sham) and ovariectomized (ovx) mice.* ***(A)*** *The uteri of sham and ovx mice. Vaginal smears from sham* ***(B)*** *and ovx* ***(C)*** *mice.*

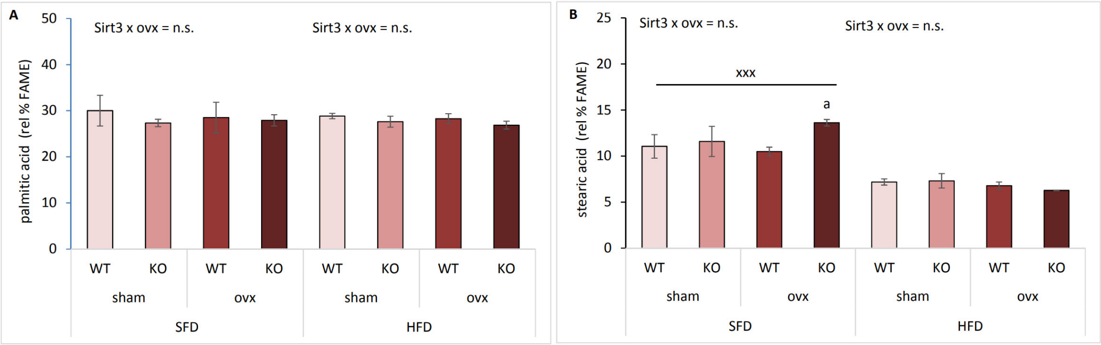

Figure S2. *Graphical display of hepatic SFA content in sham and ovx Sirt3 WT and KO mice after 10 weeks of feeding with SFD or HFD.* ***(A) Palmitic acid****. SFD: no changes. HFD: no changes. SFD vs. HFD: no changes.* ***(B) Stearic acid.*** *SFD: ovx WT vs. KO ([a]p<0.001). HFD: no changes. SFD vs. HFD: [xxx]p<0.001. Data are shown as mean ± SD. N=4 mice per group.*

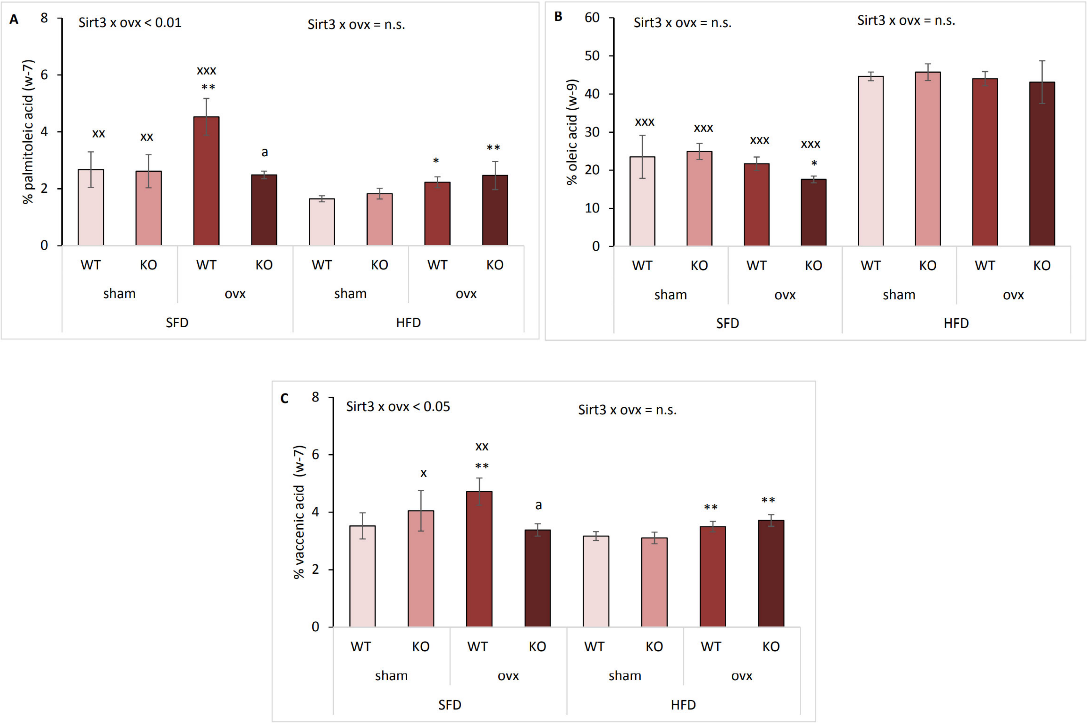

Figure S3. *Graphical display of hepatic MUFA content in sham and ovx Sirt3 WT and KO mice after 10 weeks of feeding with SFD or HFD.* ***(A) Palmitoleic acid.*** *SFD: ovx WT vs. KO ($^{a}p<0.01$); WT sham vs. ovx (**$p<0.01$). HFD: WT ovx vs. sham (*$p<0.05$); KO ovx vs. sham (**$p<0.01$). SFD vs. HFD: sham ($^{xx}p<0.01$); WT ovx ($^{xxx}p<0.001$).* ***(B) Oleic acid.*** *SFD: KO ovx vs. sham (*$p<0.05$). HFD: no changes. SFD vs. HFD: $^{xxx}p<0.001$.* ***(C) Vaccenic acid****. SFD: ovx WT vs. KO ($^{a}p<0.01$); WT sham vs. ovx (**$p<0.01$). HFD: ovx vs. sham (**$p<0.01$). SFD vs. HFD: KO sham ($^{x}p<0.05$); WT ovx ($^{xx}p<0.01$). Data are shown as mean ± SD. N=4 mice per group.*

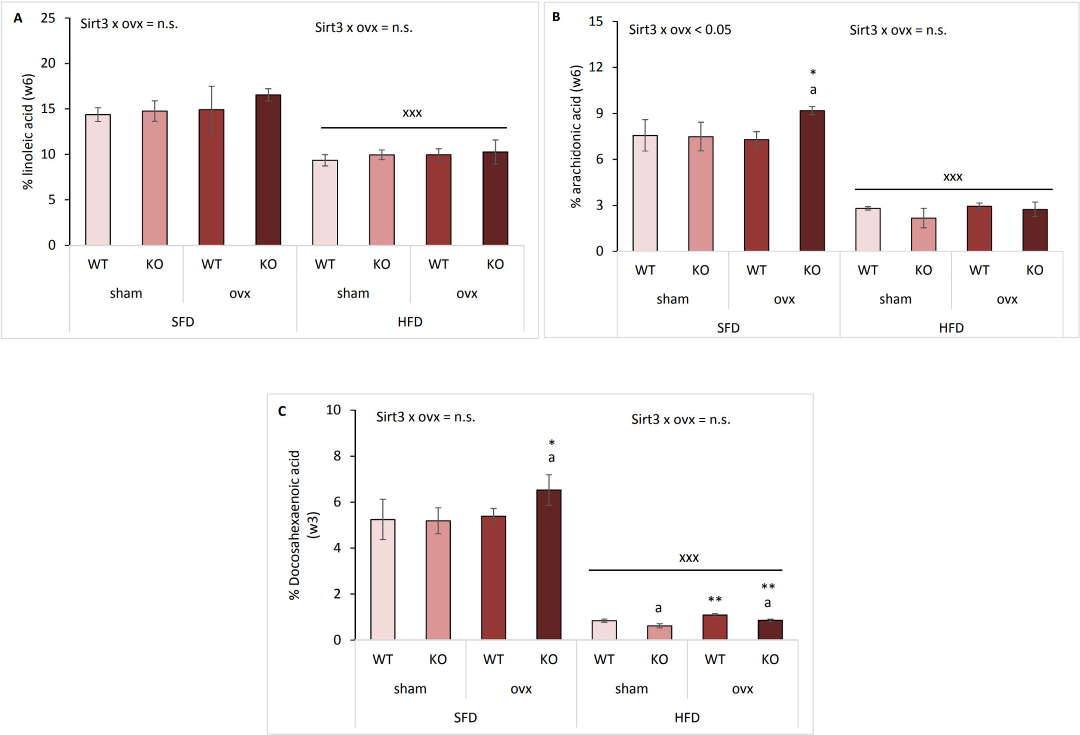

Figure S4. *Graphical display of hepatic PUFA content in sham and ovx Sirt3 WT and KO mice after 10 weeks of feeding with SFD or HFD.* ***(A) Linoleic acid****. SFD: no changes. HFD: no changes. SFD vs. HFD: $^{xxx}p<0.001$.* ***(B) Arachidonic acid.*** *SFD: ovx WT vs. KO ($^{a}p<0.01$); KO sham*

*vs. ovx (*p<0.05). HFD: no changes. SFD vs. HFD: ^{xxx}p<0.001.* ***(C) Docosahexaenoic acid**. SFD: KO sham vs. ovx (*p<0.05); ovx WT vs. KO (^{a}p<0.05). HFD: KO vs. WT (^{a}p<0.05); ovx vs. sham (**p<0.01). SFD vs. HFD: ^{xxx}p<0.001. Data are shown as mean ± SD. N=4 mice per group.*

***Table S1.** Assays (Taqman® Applied Biosystems, UK) used for the real time quantitative PCR analyses*

Gene	Assay ID	Product size (bp)
β-actin	Mm00607939_s1	115
sirt3	Mm00452131_m1	68
cyp2e1	Mm00491127_m1	83
cyp4a14	Mm00484132_m1	71
pparα	Mm00627559_m1	86
pgc1α	Mm00447183_m1	104
ho-1	Mm00516007_m1	92

***Table S2.** Antibodies used in this study for the Western blot analyses*

Antibody	Dilution	Host	Manufacturer
Sirt3 (D22A3)	1:1000	Rabbit	Cell Signaling Technology, USA
Anti-rabbit (NA934)	1:5000	Goat	GE Healthcare, USA
